# Editorial: Cystic fibrosis-related diabetes

**DOI:** 10.3389/fendo.2024.1464440

**Published:** 2024-07-26

**Authors:** James A. M. Shaw

**Affiliations:** Translational and Clinical Research Institute, The Medical School, Framlington Place, Newcastle University, Newcastle-upon-Tyne, United Kingdom

**Keywords:** cystic fibrosis, diabetes, pathology, signalling, glycaemia control and treatment

Cystic fibrosis (CF) of the pancreas on *post mortem* examination resulting in death during early life through intestinal obstruction or respiratory infections was first described by Dr Dorothy Andersen in 1938 (1). Since then, incremental improvements in care and a series of therapeutic breakthroughs have enabled steadily increasing life expectancy (2). Following confirmation that the underlying cause of CF is genetic mutations leading to loss or dysfunction of the cystic fibrosis transmembrane conductance regulator (CFTR) protein, efforts have been underway to develop CFTR modulator therapies to correct this abnormality (3). Widespread implementation of highly effective modulator therapy has been transformative for a large number of individuals with CF with significant reductions in respiratory symptoms and infective exacerbations (4).

In parallel with these improvements in life expectancy and respiratory outcomes, cystic fibrosis-related diabetes (CFRD) has emerged as an increasingly prominent co-morbidity for those living with CF. Progress towards elucidating the potential causes and underlying mechanisms are reviewed in this Frontiers in Endocrinology Research Topic, together with clinical studies undertaking detailed characterisation of impaired pancreatic endocrine function. A novel approach to predict CFRD outcomes is reported in addition to assessment of the impact on blood glucose of dietary constitution and modern diabetes self-management technology.

Coderre et al., review the wide range of pathological mechanisms within the pancreas potentially contributing to progressive pancreatic endocrine dysfunction and CFRD. They highlight that CFTR is expressed early in embryonic development with ductal structural abnormality established at birth. Summarising the immunological pathways implicated in type 1, type 2 and chronic pancreatitis-related diabetes pathogenesis, they propose that exocrine pancreatic alterations in CF lead to macrophage activation and inflammation which play an important, as yet incompletely understood, role in islet dysfunction.

With a focus on preclinical models, Gál et al., review how cytokine-mediated proinflammatory pathways can drive β-cell dysfunction in chronic pancreatitis and CFRD. Cytokines including interleukin-1β and interferon-γ have been shown to inhibit insulin secretion (5). In addition, a potentially central role for TGF- β1 signalling has been identified in a murine partial pancreatic duct ligation model leading to islet β-cell epithelial-to-mesenchymal transition (6).

Malik et al., also describe the exocrine pancreas as the ‘epicentre of pancreas pathology in CF with ductal pathology being the initiating event’. Through thorough review of the published human *post mortem* literature, they present evidence for relative maintenance of overall islet mass but changes in endocrine cell ratios including decreased β-cell and increased α-cell numbers. The destructive pathology surrounding islets is associated with changes in the microenvironment including disruption of normal vascularisation and innervation. The need for further research focused on deeper characterisation of the intra- and peri-islet niche in CF is underlined.

Nielsen et al., report their original data derived from extended oral glucose tolerance tests undertaken cross-sectionally in an adult Danish cohort with CF. In parallel with delayed and then diminished insulin secretion as glucose tolerance deteriorated, proinsulin-to-insulin ratio increased in keeping with worsening β-cell stress. An association between proinsulin secretion and impaired glucagon suppression in response to elevated glucose was seen, suggesting an association between β- and α-cell dysfunction in CF.

The team led by Andrea Kelly and Michael Rickels at the University of Pennsylvania have undertaken impressive in-depth metabolic studies to gain deeper understanding of endocrine dysfunction in CF. They have used the glucose-potentiated arginine (GPA) secretion test in multiple studies to definitively evaluate β-cell functional mass. In a secondary analysis of these studies, Malik et al., report here that early phase insulin secretion derived from the much easier to perform standardised mixed meal tolerance test (MMTT) through frequent sampling particularly over the first 30 minutes accurately predicts GPA-derived measures. The authors urge incorporation of this powerful, reliable but also feasible test into large prospective studies and multi-centre clinical trials to provide deeper phenotyping of participants and greater insights into the impact of the therapeutic intervention.

Scully et al., undertook a prospective observational study evaluating the predictive value of continuous glucose monitoring in people with CF. In parallel, the group evaluated advanced glycation end-products (AGE) assessed by skin autofluorescence and report for the first time higher values in those with CFRD and those with more time spent with high glucose levels on continuous glucose monitoring (CGM) or with elevated HbA1c. AGE values predicted weight loss and FEV1 decline over the following year.

Dietetic guidance for those with CF has traditionally advocated high calorie intake with a focus on ‘energy-dense, nutrient poor’ food. Concerns are increasingly being voiced, however, regarding the potential detrimental effect on glycaemic status, particularly as long-term health and cardiovascular risk reduction comes to the fore in those thriving from the respiratory point of view and gaining weight on highly effective CFTR modulator therapy (7). In a cross-sectional study in 27 adults with CF, β-cell function was inversely associated with dietary glycaemic load and positively associated with unsaturated fat intake. Higher plant protein intake was possibly associated with reduced insulin resistance. Larger studies prospectively evaluating the impact of modifying macronutrient constituency and quality in CF are clearly merited.

In addition to CGM, insulin pump technology initially pioneered for type 1 diabetes self-management is now being considered for those with CFRD. In a non-randomised trial of sensor-augmented pump therapy in insulin-treated individuals with CFRD in Italy, Grancini et al. , showed a highly clinically significant 1% lower HbA1c in addition to increased fat-free body mass compared with those choosing to continue on multiple daily injection therapy. Multicentre randomised controlled trials evaluating hybrid closed loop CGM/insulin pump therapy in CF are currently underway internationally (8).

CFRD is justifiably becoming a major focus for CF basic and translational research. This Research Topic provides powerful evidence of the quality and depth of the inter-disciplinary work already underway and the momentum which is being gathered. This series of articles has illuminated multiple target areas where further investment offers realistic promise for rapid transformation in glycaemic outcomes in CF to complement the successes achieved in pulmonary disease.

## Author contributions

JS: Writing – review & editing, Writing – original draft, Conceptualization.
